# KIF3A binds to β-arrestin for suppressing Wnt/β-catenin signalling independently of primary cilia in lung cancer

**DOI:** 10.1038/srep32770

**Published:** 2016-09-06

**Authors:** Minsuh Kim, Young-Ah Suh, Ju-Hee Oh, Bo Ra Lee, Joon Kim, Se Jin Jang

**Affiliations:** 1 Biomedical science and engineering interdisciplinary program, KAIST, Daejeon, South Korea; 2 Asan Institute for Life Sciences, University of Ulsan College of Medicine, Asan Medical Center, Seoul, South Korea; 3 Graduate School of Medical Science and Engineering, KAIST, Daejeon, South Korea; 4 Asan Center for Cancer Genome Discovery, Department of Pathology, University of Ulsan College of Medicine, Asan Medical Center, Seoul, South Korea

## Abstract

Aberrant Wnt/β-catenin signalling is implicated in the progression of several human cancers, including non-small cell lung cancer (NSCLC). However, mutations in Wnt/β-catenin pathway components are uncommon in NSCLC, and their epigenetic control remains unclear. Here, we show that KIF3A, a member of the kinesin-2 family, plays a role in suppressing Wnt/β-catenin signalling in NSCLC cells. KIF3A knockdown increases both β-catenin levels and transcriptional activity with concomitant promotion of malignant potential, such as increased proliferation and migration and upregulation of stemness markers. Because KIF3A binds β-arrestin, KIF3A depletion allows β-arrestin to form a complex with DVL2 and axin, stabilizing β-catenin. Although primary cilia, whose biogenesis requires KIF3A, are thought to restrain the Wnt response, pharmacological inhibition of ciliogenesis failed to increase β-catenin activity in NSCLC cells. A correlation between KIF3A loss and a poorer NSCLC prognosis as well as β-catenin and cyclin D1 upregulation further suggests that KIF3A suppresses Wnt/β-catenin signalling and tumourigenesis in NSCLC.

Wnt signalling governs cell fate and proliferation during embryonic development and plays important roles in adult tissue homeostasis^1^. In the absence of Wnt, the β-catenin destruction complex, consisting of axin, adenomatous polyposis coli (APC), and glycogen synthase kinase 3β (GSK3β), inhibits β-catenin accumulation. The binding of Wnt to its receptor FZD and co-receptor LRP results in a series of phosphorylation events that transiently represses the β-catenin destruction complex via Dishevelled (DVL), stabilizing β-catenin. Transcription of Wnt target genes is activated when stabilized β-catenin enters into the nucleus and functions as a transcriptional coactivator of members of the TCF/LEF family of transcription factors^1^,^2^.

Aberrant Wnt/β-catenin signalling drives oncogenesis in several human cancers with mutations in core signalling components, such as APC and CTNNB1 (β-catenin), driving constitutive pathway activation^3^. Mutations in Wnt signalling components are rarely found in NSCLC^4^. Nevertheless, frequent overexpression of Wnt lignads (WNTs) and the Wnt components is associated with poor NSCLC prognosis^4^ and resistance of metastatic NSCLC to chemotherapy^4^,^5^. In contrast, endogenous Wnt inhibitors have been observed to be lost or downregulated in NSCLC^4^. Suppression of Wnt/β-catenin activation by either restoration of Wnt inhibitor function or depletion of Wnt or DVL arrests the proliferation and motility of NSCLC cells and increases their apoptosis^4^,^6^. These findings suggest that alterations in Wnt/β-catenin signalling substantially contribute to the aggressiveness of NSCLC.

KIF3A belongs to the kinesin family of proteins that function as a molecular motor transporting cargo along microtubules. KIF3A is best known for its role in molecule transport along the axoneme of cilia, and loss of KIF3A causes defects in cilium biogenesis^7^,^8^. Also, KIF3A forms a complex with APC and participates in the transport of APC, supporting cell migration and polarization^9^. Additionally, KIF3A constrains the activity of the Wnt/β-catenin pathway by suppressing casein kinase 1 (CK1)-dependent DVL phosphorylation, which is a key step in Wnt signalling. Loss of KIF3A in mouse embryonic fibroblasts results in constitutive DVL phosphorylation and potentiates Wnt3a-induced β-catenin stabilization^10^. However, a recent study presented contradictory results in which a knockdown of KIF3A reduced Wnt/β-catenin signalling in prostate cancer cells, whereas overexpression of KIF3A promoted it. Moreover, that study reported that KIF3A increased DVL2 phosphorylation by CK1 in prostate cancer cells^11^.

Further complexity in the relationship between KIF3A and Wnt signalling comes from the finding that primary cilia are involved in the regulation of the Wnt response. Ablation of primary cilia via deletion of ciliary or basal body genes other than KIF3A causes hyperactivation of Wnt/β-catenin signalling in response to Wnt3a stimulation^10^,^12^,^13^. Similarly, depletion of Bardet–Biedl syndrome (BBS) protein, which is a component of the basal body, causes defective proteasomal targeting and concomitant accumulation of β-catenin, which substantially increases the Wnt response^14^. However, loss of ciliogenic genes except KIF3A does not affect DVL phosphorylation^10^. Therefore, KIF3A may exert its effect on Wnt/β-catenin signalling through both cilium-dependent and -independent mechanisms^10^. Unexpectedly, an earlier study showed that the expression levels of the Wnt target gene Axin2 are unaltered in mouse embryos lacking primary cilia due to knockout of ciliogenic genes including KIF3A^15^. Thus, the significance and mechanisms of KIF3A and primary cilia in the regulation of Wnt signalling are still unclear.

In our current study, we reveal a tumour suppressor role for KIF3A as an inhibitor of the Wnt/β-catenin pathway in NSCLC cells. We provide evidence that KIF3A inhibits Wnt signalling through interaction with β-arrestin, as a non-ciliary mechanism. Importantly, KIF3A loss was correlated with increased expression of both β-catenin and cyclin D1 in clinical NSCLC samples and also associated with worse patient survival. Our results indicate that KIF3A is a class of tumour suppressor in NSCLC.

## Results

### KIF3A knockdown promotes Wnt/β-catenin signalling in lung cancer cells

**Figure 1:**
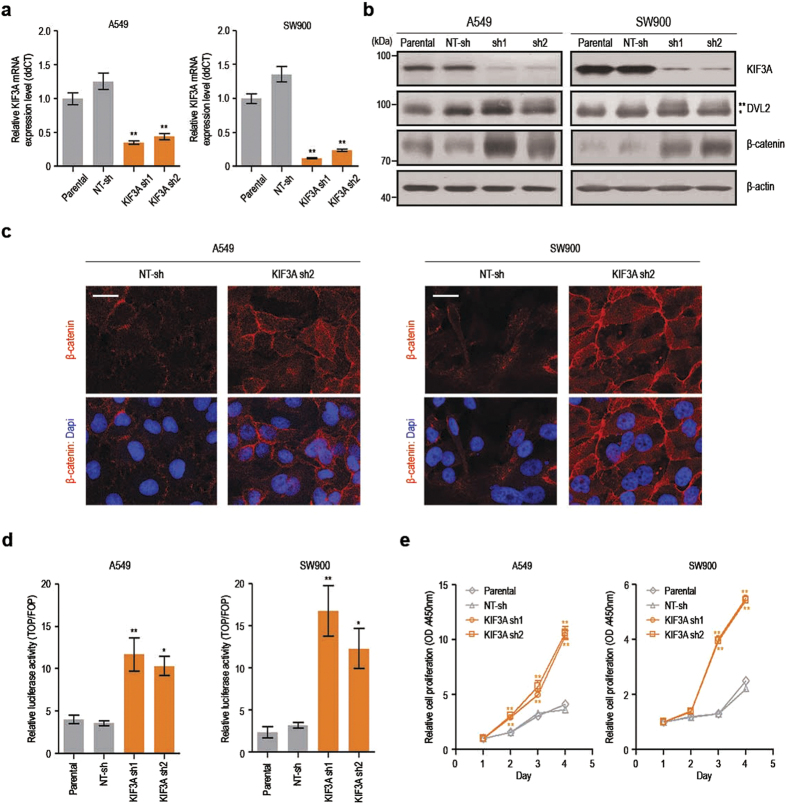
KIF3A knockdown results in hyperactivation of Wnt/β-catenin signalling. (**a**) Quantitative RT-PCR analysis confirming reduction of KIF3A transcripts in cells expressing KIF3A shRNA. β-actin was used as an internal control for quantitative RT-PCR (n = 3; error bars indicate SEM; p-value was determined by one-way ANOVA). (**b**) Immunoblot analysis showing protein levels of β-catenin and DVL2 after KIF3A knockdown. (*DVL2; **phosphorylated-DVL2). β-actin was used as a loading control. (**c**) Immunofluorescence images showing the expression of β-catenin (scale bar: 20 μm). Nuclei (blue) were stained with DAPI. (**d**) SuperTOPFlash reporter assay to determine Wnt/β-catenin signalling activity. Cells were transfected with pM50 Super 8xTOPFlash or pM51 Super 8xFOPFlash for 24 h. SuperTOPFlash reporter activity was normalized by Renilla luciferase (RL) activity. Relative luciferase reporter activity was calculated by dividing TOP/RL ratio by FOP/RL ratio (n = 5; error bars indicate SEM; p-value was determined by one-way ANOVA). (**e**) Proliferation analysis of A549 and SW900 cells transfected with NT-sh, KIF3A sh1 or sh2. Cell proliferation was measured by CCK assay for 4 days (n = 8; error bars indicate SEM; p-value was determined by one-way ANOVA). *P < 0.05; **P < 0.01. Full-length blot is shown in the supplementary information, and the gels have been run under the same experimental conditions.

To investigate the role of KIF3A in Wnt pathway regulation in lung cancer, we established A549 and SW900 NSCLC cell lines stably expressing small hairpin RNAs (shRNAs) specific for KIF3A. Two distinct KIF3A shRNAs were used to minimize sequence-dependent off-target effects, and cell lines harbouring non-target shRNA (NT-sh) were also generated. Our real-time RT-PCR and western blot analyses showed that both KIF3A shRNA1 and shRNA2 decreased the level of KIF3A mRNAs and proteins (Fig. 1a,b). Next, we examined the β-catenin protein level, which is an indicator of the activation status of Wnt/β-catenin signalling, in cells transfected with KIF3A shRNAs. As shown in Fig. 1b, KIF3A knockdown markedly elevated β-catenin levels in both A549 and SW900 cells. DVL2 is known to be phosphorylated in response to WNTs, and phosphorylated DVL2 stabilizes cytosolic β-catenin by inhibiting the β-catenin destruction complex^16^. As expected, elevated β-catenin levels were accompanied by increased DVL2 phosphorylation in A549 and SW900 cell lines depleted of KIF3A (Fig. 1b). These results suggest that KIF3A is involved in the regulation of Wnt signalling in A549 and SW900 cells. We also performed immunofluorescence analyses to visualize the localization of β-catenin. KIF3A knockdown in A549 and SW900 cells increased β-catenin immunoreactivity in the nucleus as well as in the cytosol and cell junctions at the plasma membrane (Fig. 1c).

Next to test if KIF3A knockdown affects β-catenin–dependent transcriptional activity, we performed a SuperTOPFlash reporter assay. As expected, KIF3A knockdown increased the transcriptional activity of β-catenin (Fig. 1d). Upregulation of β-catenin transcriptional activity is closely associated with increased cell proliferation^16^,^17^. Thus, we investigated the impact of KIF3A depletion on the proliferation of the NSCLC cell lines. In accordance with the elevated β-catenin levels and activities, cells depleted of KIF3A exhibited an increased cell proliferation rate (Fig. 1e).

### KIF3A knockdown affects malignant potential of lung cancer cells

**Figure 2:**
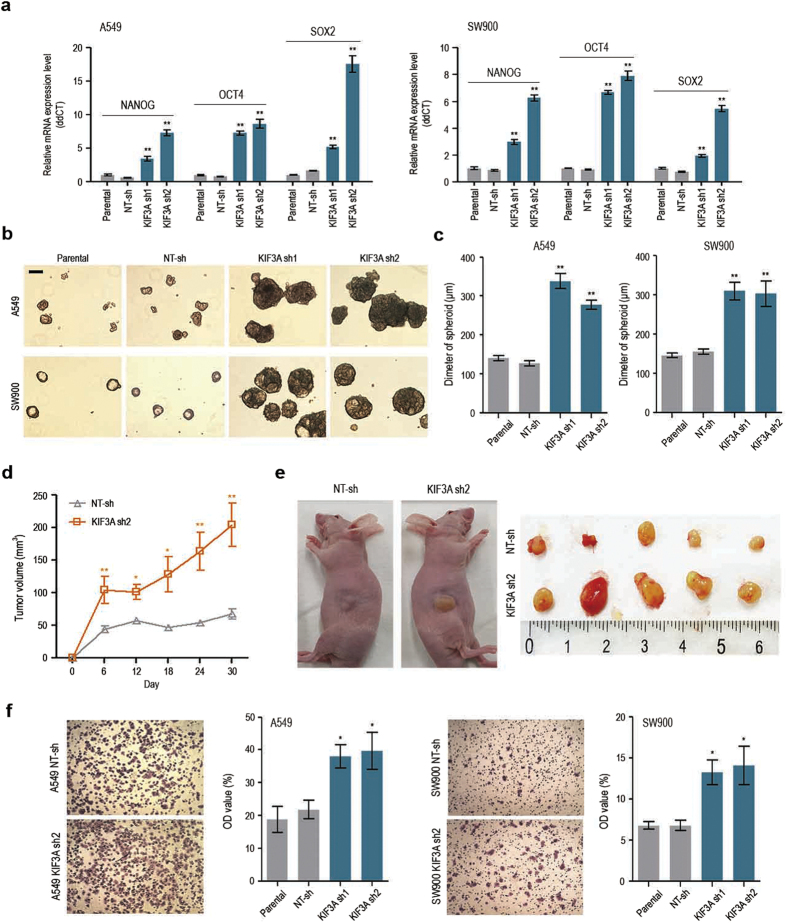
KIF3A knockdown promotes malignant phenotypes in NSCLC cell lines. (**a**) Quantitative RT-PCR analysis showing the expression of stemness markers, NANOG, OCT4 and SOX2. GAPDH was used as an internal control (n = 3; error bars indicate SEM; p-value was determined by two-way ANOVA). (**b**) Sphere-formation assay in cells transfected with KIF3A sh1 or sh2. Cells were cultured in 6 well ultra-low attachment plate at a density of 3 × 103 cells per well for 7 days. (**c**) Measurement of the diameter of spheres illustrated in B, using image j (n = 5; error bars indicate SEM; p-value was determined by one-way ANOVA). (**d**,**e**) Growth of tumours derived from SW900 cells transfected with NT-sh or KIF3A sh2. NT-sh and KIF3A sh2 cells were separately implanted into nude mice through subcutaneous injection. Tumour volume was measured every 6 days. Tumour growth curve (**d**) and photographs of excised tumours (**e**) indicating that the mice injected with KIF3A sh2 cells had larger tumours than mice injected with NT-sh cells (n = 5). (**f** ) Transwell chamber assay in cells transfected with KIF3A shRNA (n = 3; error bars indicate SEM; p-value was determined by one-way ANOVA). *P < 0.05; **P < 0.01.

Cancer stem-like cells (CSCs), also called tumour-initiating cells, are a subset of tumour cells characterized by a capacity for self-renewal and high tumourigenic potential. NANOG, OCT4, and SOX2, which are transcription factors essential for maintaining the pluripotency of normal stem cells, control tumour initiation and malignancy in several types of cancers, including lung adenocarcinoma^18^,^19^. Additionally, Wnt/β-catenin signalling has been implicated in stemness promotion in both normal stem cells and CSCs^2^. We analysed the expression levels of NANOG, OCT4, and SOX2 using quantitative RT-PCR. As shown in Fig. 2a, knockdown of KIF3A resulted in an upregulation of all three stemness markers in A549 and SW900 cells. To gain insights into the functional consequences of stemness gene expressions, we performed a sphere-forming assay, which is one of the methods used to demonstrate the presence of stem/progenitor cells in a population of normal or tumour cells^20^. In serum-free, non-adherent conditions, KIF3A-depleted cells formed significantly bigger spheres than parental and NT-sh–containing cells (Fig. 2b,c). These results suggest that KIF3A depletion promotes the acquisition of stemness in NSCLC cell lines.

Next, to demonstrate the tumourigenicity of KIF3A-depleted cells, we implanted the cells into nude mice through subcutaneous injection, and measured the tumour volume. Although injected control SW900 cells formed a tumour mass, the tumours did not grow in size for at least 1 month (Fig. 2d,e). In contrast, SW900 cells depleted of KIF3A formed a larger initial tumour mass, and the tumour volume doubling time was less than 3 weeks (Fig. 2d,e). Elevated expression of OCT4 and NANOG promotes the epithelial–mesenchymal transdifferentiation of A549 cells and also increases their metastatic abilities in xenograft models^18^. To test the effect of KIF3A knockdown on cell migration, we performed a migration assay using Transwell chambers. Compared with control cells, cell migration was accelerated in cells expressing KIF3A shRNA (Fig. 2f). Taken together, these results indicate that loss of KIF3A aggravates tumour growth and increases tumour cell migratory behavior.

**Figure 3:**
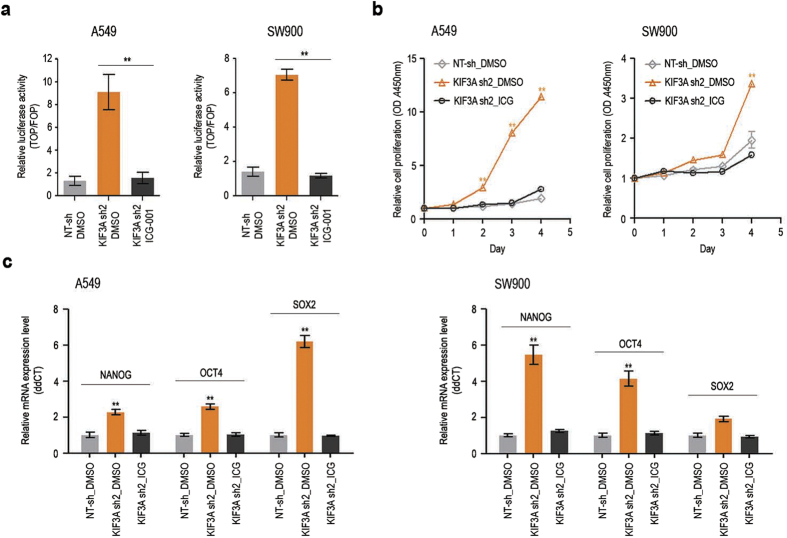
Inhibition of Wnt/β-catenin signalling suppresses KIF3A-knockdown phenotypes. (**a**) ICG-001-mediated suppression of Wnt/β-catenin hyper activation in KIF3A-depleted cells. Cells were transfected with TOPFlash or FOPFlash, and treated with DMSO or ICG-001 (7 μM) for 24 h. Luciferase reporter activity was calculated by dividing TOP/RL ratio by FOP/RL ratio (n = 5, error bars indicates SEM; p-value was determined by unpaired t-test). (**b**) Suppression of the active proliferation of KIF3A-depleted cells by ICG-001. Cell proliferation was measured by CCK assay for 4 days (n = 8; error bars indicate SEM; p-value was determined by one-way ANOVA). (**c**) Quantitative RT-PCR analysis of stemness markers, Nanog, OCT4 and SOX2 after ICG-001 treatment (n = 3; error bars indicate SEM; p-value was determined by two-way ANOVA). GAPDH was used as an internal control. *P < 0.05; **P < 0.01.

Activation of the Wnt/β-catenin pathway is likely to underlie the CSC-like malignant properties observed in KIF3A-depleted cells. To confirm this idea, we investigated the effect of the Wnt pathway inhibitor ICG-001 on cell proliferation and stemness marker expression. ICG-001 blocks the interaction of CREB-binding protein with β-catenin and thus inhibits the transcriptional co-activator function of β-catenin^21^. First, we evaluated the ability of ICG-001 to inhibit Wnt signalling in A549 and SW900 cells using a SuperTOPFlash reporter assay. As expected, β-catenin–dependent luciferase expression was suppressed by ICG-001 in KIF3A-depleted cells (Fig. 3a). We next tested if ICG-001 could inhibit the elevated cell growth induced by KIF3A knockdown. As shown in Fig. 3b, the exponential growth of both A549 and SW900 cells depleted of KIF3A was efficiently blocked by ICG-001 treatment. Moreover, ICG-001 suppressed the upregulation of the stemness markers in both cell lines (Fig. 3c). In addition, to clarify that Wnt/β-catenin signalling regulates CSC-like malignant potential in these cells, we induced Wnt/β-catenin signalling by treating GSK3 inhibitor LiCl and investigated expression of stemness markers in NT-sh cells. NT-sh cells treated with LiCl showed elevated expression of NANOG and SOX2 (Supplementary Fig. 1). These results suggest that KIF3A influences the proliferation and tumorigenicity of lung cancer cells via its role in the regulation of Wnt/β-catenin signalling.

### Wnt/β-catenin signalling is not affected by primary cilia in lung cancer cells

**Figure 4:**
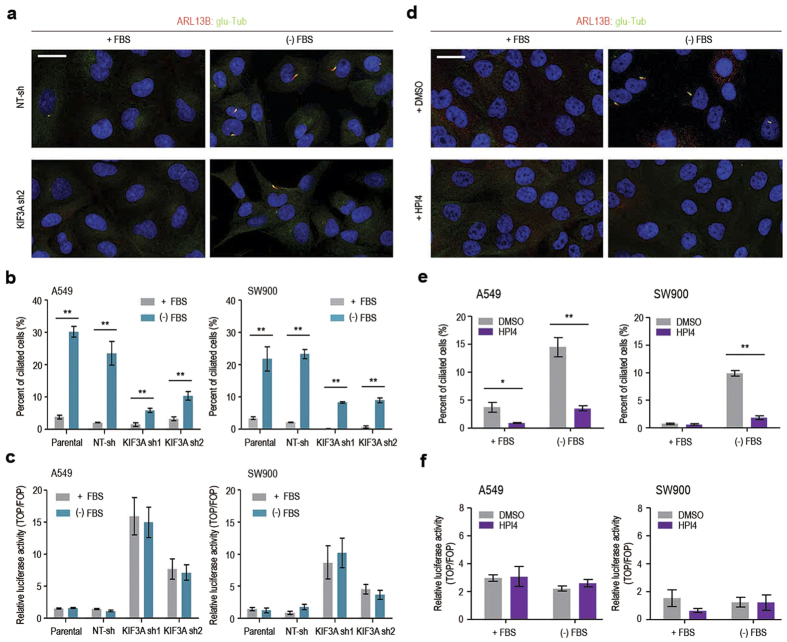
Primary cilia do not influence Wnt/β-catenin signalling in NSCLC cell lines. (**a**) Immunofluorescence staining of primary cilia in A549 cells transfected with NT-sh or KIF3A sh2 (scale bar: 20 μm). (−) FBS indicates serum starvation for 48 h. Nuclei (blue) were stained with DAPI. (**b**) Quantitative analysis of the experiment shown in A (n = 3; error bars indicate SEM; p-value was determined by unpaired t-test). (**c**) SuperTOPFlash reporter assay to compare Wnt activity in the presence and absence of FBS for 48 h. Luciferase reporter activity was calculated by dividing TOP/RL ratio by FOP/RL ratio. (n = 5; error bars indicate SEM; p-value was determined by unpaired t-test). (**d**) Immunofluorescence staining of primary cilia in SW900 cells treated with DMSO or HPI4 (scale bar: 20 μm). Cells were treated with DMSO or HPI4 (20 μM) in the presence or absence for 24 h. (**e**) Quantitative analysis of the experiment shown in C (n = 3; error bars indicate SEM; p-value was determined by unpaired t-test). (**f** ) SuperTOPFlash reporter assay to examine the effect of HPI4 on Wnt/β-catenin signalling activity. Luciferase reporter activity was calculated by dividing TOP/RL ratio by FOP/RL ratio (n = 5; error bars indicate SEM; p-value was determined by unpaired t-test). *P < 0.05; **P < 0.01.

Although there are some contradictory results in the literature^12^,^13^,^14^,^15^, primary cilia have been implicated in the negative regulation of Wnt/β-catenin signalling in both developing mouse embryos and HEK293T cells. KIF3A plays an essential role in the biogenesis of primary cilia, and knockdown or knockout of the KIF3A gene has been widely used to analyse the function of primary cilia^22^,^23^,^24^. Thus, we examined the possibility that KIF3A knockdown indirectly affects Wnt/β-catenin signalling through its role in the assembly of primary cilia in lung cancer cells. As shown in Fig. 4a,b, only a few ciliated cells were observed in cell cultures maintained in 10% FBS, whereas ciliated cell frequencies were significantly increased by serum starvation in both A549 and SW900 cells. This observation confirms previous findings that ciliogenesis is linked to cell cycle quiescence^25^,^26^. KIF3A depletion reduced ciliated cell numbers (Fig. 4a,b). However, the proportion of ciliated cells was significantly increased by serum starvation, even after KIF3A knockdown, possibly due to incomplete gene silencing. Next, to evaluate the correlation between ciliation and β-catenin–dependent transcriptional activity, we performed a SuperTOPFlash reporter assay. Importantly, β-catenin–dependent transcription was not noticeably affected by serum starvation, which exerted a clear positive effect on cilium assembly (Fig. 4c). This suggests that Wnt/β-catenin signalling is independent of primary cilia in A549 and SW900 cells.

To further demonstrate cilium-independent regulation of Wnt signalling in lung cancer cells, we examined the effect of HPI4, a small molecule inhibitor of ciliogenesis^27^, on β-catenin-dependent transcriptional activity. As expected, HIP4 treatment inhibited ciliogenesis induced by serum starvation in both cell lines (Fig. 4d,e). However, suppression of ciliogenesis did not lead to a corresponding increase in β-catenin-dependent transcriptional activity (Fig. 4f). HPI4 did not induce apoptosis at the same dose in these cells, excluding the possibility that HPI4 toxicity suppressed reporter gene expression (Supplementary Fig. 2). Together, these results suggest that the activation of Wnt/β-catenin signalling in KIF3A-depleted cells is not a secondary consequence of disrupted ciliogenesis.

### KIF3A binds to β-arrestin for inhibiting autocrine Wnt/β-catenin signalling

**Figure 5:**
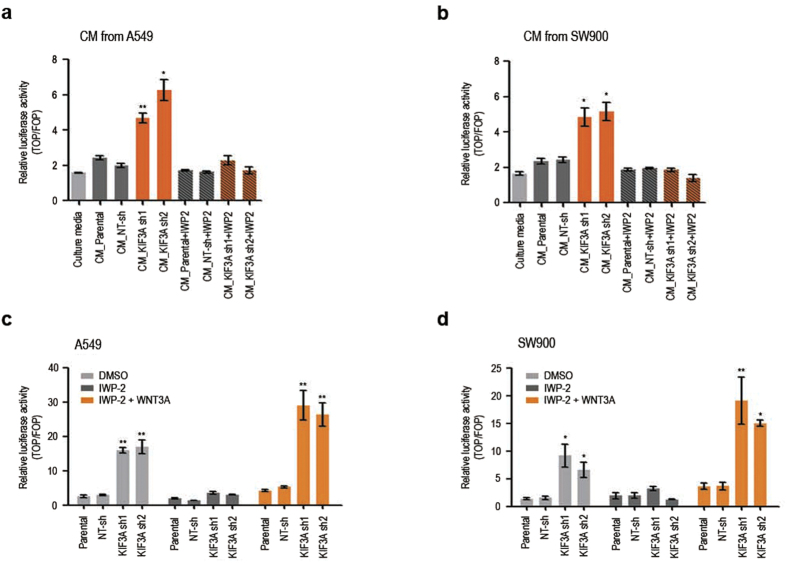
KIF3A knockdown promotes both ligand secretion and intracellular signalling of the Wnt/β-catenin pathway in NSCLC cell lines. (**a**,**b**) SuperTOPFlash reporter assay to examine the effect of KIF3A knockdown on the secretion of WNTs by A549 and SW900 cell lines. HEK293T cells were culture in the presence of conditioned media (CM) from A549 and SW900 cell lines for 48 h, and the activity of Wnt/β-catenin signalling in HEK293T cells was assessed by luciferase reporter activity. To block WNT secretion during the production of CM, A549 and SW900 cells were treated with IWP-2 (30 μM). Luciferase reporter activity was calculated by dividing TOP/RL ratio by FOP/RL ratio (n = 3, error bars indicate SEM; p-value was determined by unpaired t-test). (**c**,**d**) SuperTOPFlash reporter assay to examine the effect of KIF3A knockdown on signalling pathway activation by autocrine or exogenous WNTs in A549 and SW900 cell lines. A549 and SW900 cells were incubated with DMSO or IWP-2 (30 μM) for 48 h, and then treated with BSA or WNT3A (100 ng/ml) for additional 24 h. Luciferase reporter activity was calculated by dividing TOP/RL ratio by FOP/RL ratio (n = 3; error bars indicate SEM; p-value was determined by one-way ANOVA). *P < 0.05; **P < 0.01.

Autocrine signalling has been shown to contribute to the activation of the Wnt/β-catenin pathway in NSCLC^28^. Concordantly, Wnt/β-catenin pathway activation in KIF3A depleted cells occurred without adding exogenous WNTs in culture media (Fig. 1). Therefore, we examined whether Wnt/β-catenin signalling induced by KIF3A knockdown is ascribed to a promotion of autocrine WNT secretion. As shown in Fig. 5a,b, conditioned media (CM) produced by parental A549 and SW900 slightly increased β-catenin–dependent transcriptional activity in HEK293T cells. Notably, HEK293T cells treated with CM from KIF3A depleted cells showed significantly increased Wnt/β-catenin pathway activation. This result implies that WNT secretion by A549 and SW900 is promoted by KIF3A knockdown. To validate this experiment, we tested the effect of porcupine inhibitor IWP-2, which is known to block WNT secretion. As expected, HEK293T cells cultured in CM from A549 or SW900 cells treated with IWP-2 did not show a clear induction of Wnt/β-catenin signalling. The activity of IWP-2 was confirmed by demonstrating IWP-2-mediated reduction of LRP and phosphorylated LRP expression (Supplementary Fig. 3). Next, to test whether KIF3A knockdown causes increased cellular responsiveness to WNTs as well as elevated WNT secretion, we treated exogenous WNT3A to cells depleted of KIF3A while blocking WNT secretion by IWP-2 (Fig. 5c,d). Interestingly, when treated with the same concentration of WNT3A, KIF3A depleted cells showed significantly higher levels of Wnt/β-catenin pathway activation, as compared with the activation of control cells (Fig. 5c,d). This result shows that KIF3A knockdown facilitate the activation of Wnt/β-catenin signalling. Together, our findings suggest that KIF3A plays a role in controlling both ligand secretion and intracellular signal transduction of the Wnt/β-catenin pathway, suppressing an autocrine Wnt signalling loop in NSCLC.

**Figure 6:**
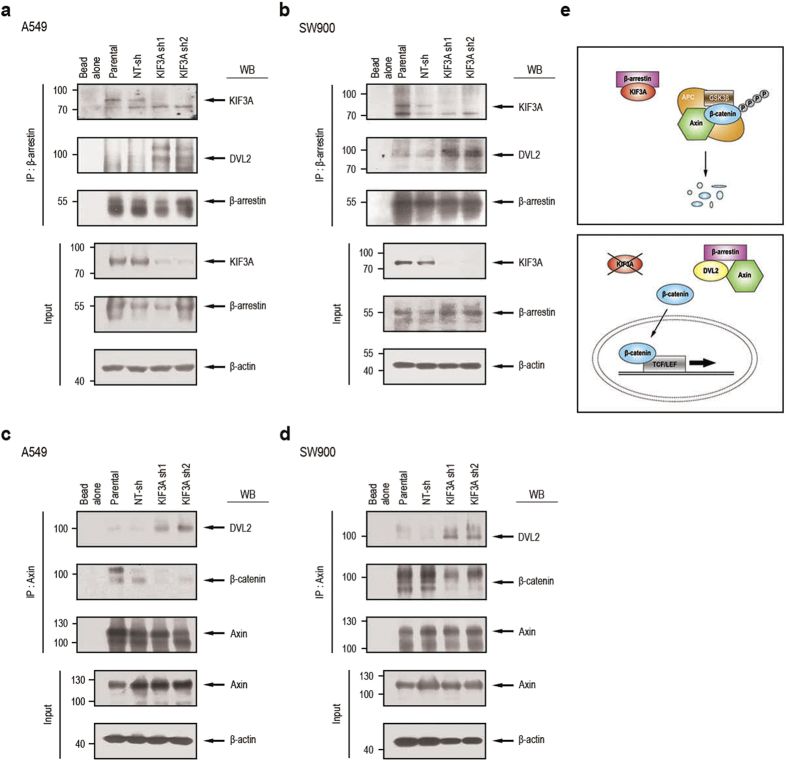
KIF3A forms a complex with β-arrestin to suppress Wnt/β-catenin signalling. (**a**,**b**) Immunoprecipitation (IP) confirming KIF3A binding to β-arrestin in the A549 and SW900 cell groups. The following groups were studied: bead alone, parental cells, NT-sh cells, KIF3A sh1 cells, and KIF3A sh2 cells. IP was performed with anti-β-arrestin antibody in cell lysates from the A549 group (**a**) and SW900 group (**b**). After the cell extracts were precipitated with anti-β-arrestin antibodies, western blotting analysis was performed with the indicated antibodies (anti-KIF3A and DVL2 antibodies). (**c**,**d**) IP showing the formation of complexes with axin. IP was performed with anti-axin antibody in cell lysates from the A549 group (**c**) or SW900 group (**d**). Axin complexes were confirmed by western blotting with the indicated antibodies (anti-DVL2 and β-catenin antibodies). (**e**) A schematic illustration of the role of KIF3A in NSCLC. As KIF3A binds to β-arrestin, the destruction complex composed of APC, axin, and GSK3β, binds to β-catenin. Eventually, β-catenin is degraded. However, when KIF3A is decreased, β-arrestin is released from binding to KIF3A. Released β-arrestin binds to DVL2 and axin, resulting in degradation of the destruction complex. Thus, β-catenin is stabilized and enters the nucleus to start transcription. Full-length blot is shown in the supplementary information, and the gels have been run under the same experimental conditions.

We next investigated the mechanism of KIF3A-mediated inhibition of Wnt signal transduction. Previous proteomic analyses revealed that KIF3A physically interacts with β-arrestin^29^, which is a multifunctional scaffold protein known to function as a necessary component of Wnt signalling^30^. β-arrestin preferentially binds to phosphorylated DVL, and this interaction appears to enhance Wnt/β-catenin signalling^30^,^31^. To test if KIF3A influences the interaction between β-arrestin and DVL, we performed co-immunoprecipitation analyses. As expected, KIF3A was co-precipitated with antibodies specific for β-arrestin (Fig. 6a,b). Interestingly, we observed an enhanced interaction between β-arrestin and DVL2 in lysates prepared from A549 and SW900 cells depleted of KIF3A (Fig. 6a,b). This result suggests that KIF3A forms a complex with β-arrestin and restrains β-arrestin activation of DVL.

β-arrestin forms a ternary complex with DVL and axin and thus plays a role in the inactivation of the β-catenin destruction complex^30^,^31^. Accordingly, we speculated that the β-catenin destruction complex would be formed in the presence of KIF3A, whereas the DVL/β-arrestin/axin complex would be formed in the KIF3A-depleted condition. To test this idea, we performed co-immunoprecipitation analyses using anti-axin antibody. In both parental and control shRNA-transfected cells, the interaction between axin and β-catenin was clearly detectable, whereas axin-DVL2 interaction was relatively low (Fig. 6c,d). In contrast, in cells depleted of KIF3A, axin-DVL2 interaction was increased, whereas axin binding to β-catenin was decreased (Fig. 6c,d). Our results suggest that β-arrestin released from KIF3A assembles a complex with DVL2 and axin that interferes with the formation of the β-catenin destruction complex and thus stabilizes β-catenin (Fig. 6e).

### Inverse correlation between KIF3A and β-catenin expression in NSCLC tissue

**Figure 7:**
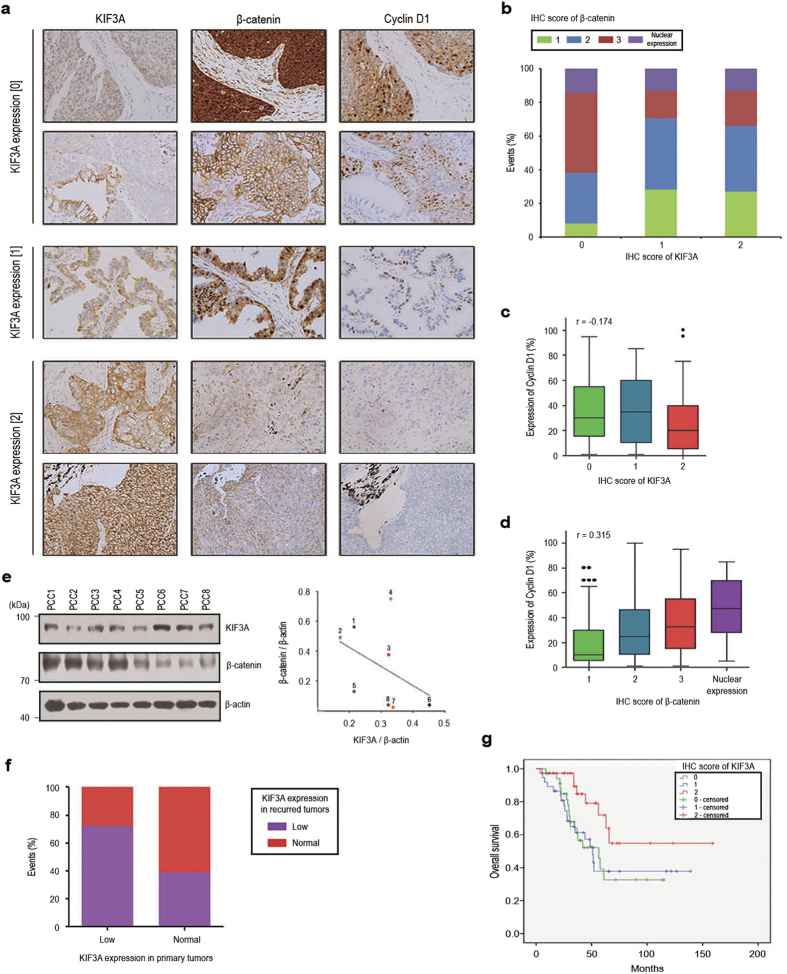
Inverse correlation between KIF3A levels and Wnt/β-catenin signalling in NSCLC patient samples. (**a**) Immunohistochemical analysis of KIF3A, β-catenin, and cyclin D1 staining in lung cancer tissues from NSCLC patients. (**b**) Summary graph describing the inverse correlation between KIF3A and β-catenin illustrated in A. For the expression of KIF3A, the number of samples expressing β-catenin with a score of 1 or 2 (weak expression level) increased. All expression scores were obtained from the immunohistochemistry results. (**c**) Quantification of immunohistochemistry results showing an inverse correlation between KIF3A and Cyclin D1 levels (r and p-value was determined by Pearson’s correlation analysis: r = −0.174, P = 0.008). (**d**) Quantification of immunohistochemistry results showing a correlation between β-catenin and Cyclin D1 levels (r and p-value was determined by Pearson’s correlation analysis: r = 0.315, P < 0.005). (**e**) Western blotting analysis showing an inverse correlation between KIF3A and β-catenin in 8 primary lung cancer cells and a quantification graph of western blotting. KIF3A and β-catenin expression levels were normalized to those of β-actin. (**f** ) Quantification of the change in KIF3A expression levels between primary and recurred tumours. Based on KIF3A score shown in Dataset EV1, the samples were classified as KIF3A-low (score 0 and 1) and normal (score 2) (KIF3A-low primary tumour n = 72; KIF3A-normal primary tumour n = 38). (**g**) Kaplan-Meier overall survival curves for 110 NSCLC patients according to KIF3A expression (p-value was determined by log-rank test: P < 0.05). Full-length blot is shown in the supplementary information, and the gels have been run under the same experimental conditions.

Hyperactivation of Wnt/β-catenin signalling has been implicated in the development of various cancers. Therefore, our findings so far suggest that KIF3A might play a role as a tumour suppressor. To validate the potential tumour suppressor function of KIF3A in NSCLC, we first examined the expression of KIF3A and β-catenin in 233 tumour tissue samples from 110 NSCLC patients who had multiple synchronous or metachronous tumours. Immunohistochemically stained samples were categorized into three groups (0, 1, and 2) according to the estimated KIF3A expression levels in the tumour cells compared with the expression in normal bronchial epithelial cells (Fig. 7a and Supplementary Table S1). Next, we categorized the same samples according to the estimated relative β-catenin expression levels, as well as its nuclear translocation (Supplementary Table S1). In accordance with *in vitro* data, there was an inverse correlation between KIF3A and β-catenin expression in the NSCLC samples (Fig. 7a,b). We examined the expression of Cyclin D1 as a downstream mediator of Wnt/β-catenin-dependent cell proliferation^4^,^16^. As shown in Fig. 7a,d, there was a clear positive correlation between β-catenin levels and the proportion of cells expressing cyclin D1 in the NSCLC samples. Importantly, we observed a decrease in cyclin D1-positive cell numbers in tumour samples expressing higher levels of KIF3A (Fig. 7a,c). These results suggest that KIF3A negatively regulates Wnt/β-catenin-dependent Cyclin D1 expression. Moreover, we confirmed the inverse correlation in 8 patient-derived primary lung cancer cell lines, using western blot analysis (Fig. 7e and Supplementary Table S2). Next, we compared KIF3A expression between primary tumours and recurred tumours. Interestingly, KIF3A expression pattern of primary tumours was mostly sustained after post-surgical tumour recurrence (Fig. 7f). This data support that the loss of KIF3A is a constitutive change providing certain survival advantages to cancer cells in a subset of NSCLC. Finally, we found that downregulation of KIF3A expression was associated with worse overall survival of patients who had multiple synchronous or metachronous NSCLC (Fig. 7g). Taken together, our results indicate that KIF3A plays a role as a tumour suppressor, inhibiting Wnt/β-catenin signalling in NSCLCs.

## Discussion

Deregulated expression of Wnt pathway components are frequently observed in NSCLCs^4^, and elevated Wnt/β-catenin signalling is thought to contribute to the tumourigenic process and drug resistance^32^,^33^. In the present study, we showed that KIF3A is involved in the negative regulation of Wnt/β-catenin signalling in NSCLC cell lines. KIF3A appears to form a complex with β-arrestin and restrict its interaction with DVL2 and axin, facilitating the formation of the β-catenin destruction complex. The accumulation of β-catenin in KIF3A-depleted cells results in accelerated cell proliferation and migration, which explains the elevation of their tumourigenic potential in mouse xenografts.

Growing evidence supports the involvement of stemness pathways in tumour progression, metastasis, and drug resistance^20^,^34^,^35^. Gene expression signatures of embryonic stem cells (ESCs) are characterized by upregulation of transcriptional targets of stemness factors such as OCT4, NANOG, and SOX2. Interestingly, the embryonic stem cell-like signature is detected in histologically poorly differentiated lung adenocarcinoma. Furthermore, triple positivity of OCT4/NANOG/SLUG has been associated with a poorer prognosis in lung adenocarcinoma^18^. In addition, ectopic expression of OCT4 and NANOG increases the tumour-initiating capability of lung adenocarcinoma cells, whereas OCT4/NANOG knockdown blocks the tumourigenic and metastatic ability of adenocarcinoma cells in mouse xenografts^18^. Although the Wnt/β-catenin signalling pathway promotes self-renewal in mouse ESCs^36^, the epigenetic mechanisms regulating the expression of the stemness factors in lung cancer are still poorly characterized. Because both WNTs and receptors are frequently upregulated in NSCLCs^4^,^28^,^37^, autocrine Wnt/β-catenin signalling may promote the expression of stemness factors in NSCLC. Our present results further support the link between Wnt/β-catenin signalling and stemness in NSCLC cells. Depletion of KIF3A increased both autocrine Wnt/β-catenin signalling and stemness factor expression. Moreover, inhibition of Wnt/β-catenin signalling in KIF3A-depleted cells blocked the expression of stemness factors. It is likely that KIF3A functions to limit the number of cancer cells acquiring stemness within the tumour mass through its inhibitory role in autocrine Wnt/β-catenin signalling.

Primary cilia can influence multiple signalling pathways that are implicated in cancer, including the Wnt^13^,^38^, and Hedgehog pathways^39^,^40^. Primary cilia have been observed in medulloblastoma driven by hyperactivation of Wnt or Hedgehog signalling^41^. Interestingly, primary cilia exert either positive or negative effect on the formation of medulloblastoma and basal cell carcinoma, depending on the initiating oncogenic mutation^39^,^41^. A significant reduction in ciliated cell frequency is a common feature of several types of cancer, including pancreatic cancer^42^, breast cancer^43^,^44^,^45^, prostate cancer^38^ and melanoma^46^,^47^. However, the impact of ciliary loss on how cancer cells respond to Wnt or Hedgehog ligands has not been clearly defined. It has been suggested that KIF3A provides a brake to the Wnt/β-catenin pathway in ciliated mammalian cells through both cilium-dependent and cilium-independent mechanisms^10^,^11^. Our present results do not support the cilium-dependent suppression of Wnt/β-catenin signalling in NSCLC cells. First, serum starvation-mediated induction of ciliogenesis in NSCLC cells was not accompanied by changes in β-catenin–dependent transcriptional activity. Moreover, inhibition of ciliogenesis by HPI4 did not affect Wnt/β-catenin signalling in NSCLC cells. Importantly, loss of Kif3a in actively proliferating NSCLC cells, most of which are non-ciliated, caused β-catenin stabilization as well as β-catenin–dependent transcriptional activation. Although our study does not exclude a potential role for primary cilia in NSCLC formation, KIF3A is likely to suppress autocrine Wnt/β-catenin signalling in a primary cilium-independent manner in NSCLC cells. The consequence of cilium ablation might be influenced by various cellular contexts, including gene mutations driving oncogenic signalling pathways.

The arrestin family of proteins has been identified as mediators of internalization and downregulation of GPCRs. In addition, arrestins can interact with other classes of cell surface receptors and also function as intracellular scaffolds and signalling intermediates^48^. β-arrestin is involved in the formation of signalling endosomes in β-catenin-independent Wnt signalling^31^. Moreover, β-arrestin plays a positive role in Wnt/β-catenin signal transduction through its interaction with phosphorylated DVL and axin^30^. Our current experimental results showed that KIF3A binds to β-arrestin and suppresses its inhibitory effect on the formation of the β-catenin destruction complex. Even though previous studies showed that KIF3A regulates Wnt/β-catenin signalling pathway, it had been unsolved how KIF3A mediates Wnt/β-catenin signalling. However, in this paper, we clearly demonstrated the role and mechanism of KIF3A in NSCLC cells. Interestingly, a previous study identified KIF3A as a binding partner of β-arrestin^49^. In ciliated NIH3T3 cells, the KIF3A/β-arrestin complex appears to mediate the transport of the Hedgehog pathway effector Smo to primary cilia, where Smo activates Gli transcription factors^49^. Hedgehog signalling promotes the proliferation of cerebellar granule neuron precursors and hyperactive Smo can initiate medulloblastoma formation^41^. Deletion of the KIF3A gene from established medulloblastoma in mice causes cell growth arrest and tumour regression^50^. Together, these findings raise the possibility that KIF3A coordinates the balance between Wnt and Hedgehog signalling activities. This possibility is worth exploring in the future.

A previous study using genetic modification of β-catenin showed that stabilization of β-catenin alone in the bronchiolar epithelium of the adult mouse lung does not promote tumour development, but dramatically accelerates lung tumorigenesis induced by a constitutively active Kras mutation^33^. Coactivation of Wnt/β-catenin and oncogenic Kras appears to cause transdifferentiation of bronchiolar epithelium into highly proliferative distal progenitors of embryonic lung. Thus, it seems likely that KIF3A knockdown-induced β-catenin stabilization in NSCLC cells synergizes with oncogenic mutations of the cell lines to induce phenotypic switch to more aggressive states. We observed variable levels of KIF3A expression in NSCLC patient samples. Genetic or epigenetic changes occurring in individual patients may determine the dependency of cancer cells on Wnt/β-catenin activation. Notably, patients who exhibited low KIF3A expression levels tended to maintain low KIF3A levels, even after recurrence. This suggests that KIF3A loss provides a certain selective advantage for the survival of cancer.

In conclusion, the KIF3A/β-arrestin complex restrains Wnt/β-catenin pathway activation. Moreover, KIF3A is a potential tumour suppressor in NSCLC, suppressing both autocrine Wnt/β-catenin signalling and stem cell-like properties. Importantly, the correlation between low KIF3A expression levels and a reduced overall survival of NSCLC patients indicates that the use of KIF3A as a prognostic marker would be worth exploring in the future.

## Materials and Methods

### Cell culture and drug treatment

The lung adenocarcinoma cell line A549 and lung squamous cell carcinoma cell line SW900 were purchased from American Type Culture Collection (Manassas, VA). Cells were cultured as previously described^51^. ICG-001 (Selleck, Houston, TX) was used to inhibit Wnt/β-catenin signalling, and HPI4 (Sigma, St Louis, MO) was used to inhibit ciliogenesis. LiCl (Sigma) was treated to inhibit GSK3 activation, and IWP-2 (Selleck) was treated to suppress WNT secretion.

### shRNA mediated gene silencing

KIF3A and non-target (NT) shRNA constructs were generated by Openbiosystem (GE Healthcare, Buckinghamshire, UK). The lentiviral particles expressing NT-shRNA, KIF3A shRNA1, and KIF3A shRNA2 were produced in HEK293T cells using Lenti-X™ Lentiviral Expression Systems (Clontech Laboratories, Mountain View, CA). A549 and SW900 were transduced with Lenti-X Viruses following the manufacturer’s instructions. Knockdown efficiency was determined by quantitative RT-PCR as well as western blotting 48 h after transduction. Cells harboring NT-sh or KIF3A shRNAs were selected in the media containing 2 μg/ml puromycin (Enzo Life Sciences, Inc., Farmingdale, NY).

### RT-PCR and quantitative RT-PCR

Total RNA was extracted by Trizol (Invitrogen, Carlsbad, CA) and the RNA samples were treated with DNase (Promega, Madison, WI). The samples were used to generate cDNA using a High Capacity cDNA Reverse Transcription Kit (Applied Biosystems, Carlsbad, CA). Target gene-expression levels were quantified using target specific primers. The sequence information used for RT-PCR and quantitative RT-PCR is presented in Supplementary Table S3.

### Western blotting

Harvested cultured cells were lysed in lysis buffer (Cell Signaling Technology, Danvers, MA) containing phosphatase inhibitor cocktail C (Santa Cruz, Dallas, TX). The concentration of proteins in cell lysates was quantified by the Enhanced BCA Protein Assay Kit (Pierce Biotechnology, Inc., Waltham, MA, USA) and 25 μg of proteins was loaded in each lane. Western blotting was performed as previously described^52^. Primary antibodies were incubated for overnight at 4 °C: anti-KIF3A (1:2000; Abcam, Cambridge, MA), β-catenin (1:1000; Cell Signaling Technology), DVL-2 (1:1000; Cell Signaling Technology), β-arrestin (1:1000; BioLegend Inc., San Diego, CA), axin (1:1000; Cell Signaling Technology), LRP (1:1000; Cell Signaling Technology), phosphorylated LRP (1:1000; Cell Signaling Technology) and β-actin (1:2000; Santa Cruz). Incubation with HRP-conjugated goat anti-rabbit or anti-mouse IgG secondary antibodies (1:1000; Enzo Life Sciences, Inc., Farmingdale, NY) antibodies was performed for 1 hr at room temperature. The full blots of the cropped images are presented in Supplementary Figures 4 and 5.

### Immunofluorescence

The samples were fixed in 4% PFA for 15 min, permeated with 0.5% TritonX-100/PBS for 5 min, and blocked with 3% bovine serum albumin/PBS. They were incubated with primary antibodies overnight at 4 °C. Primary antibodies were detected with Alexa Fluor 488- and 594-conjugated secondary antibodies (1:1000; Invitrogen, Carlsbad, CA) for 1 h at room temperature. Nuclei were counterstained with DAPI (1:2000; Sigma) for 15 min and imaging was visualized on Carl Zeiss fluorescence microscope (Carl Zeiss, Jena, Germany). To investigate the localization of β-catenin, cells were grown on 8-well Leb-tek II chamber slides (Thermo Fisher Scientific, Waltham, MA) at 2 × 10^4^ cells per well for 24 h. β-catenin was detected by anti-β-catenin (1:1000; Cell Signaling Technology). For analysis of primary cilia, cells were cultured in 8-well Lab-Tek II chamber slides. Primary cilia were detected by anti-ARL13B (1:1000; Proteintech, Chicago, IL) and poly-glutamylated Tubulin (1:1000; glu-Tub, Adipogen, San Diego, CA).

### TCF reporter assay

Wnt signalling activation was measured by transfection with SuperTOPFlash reporter plasmids (Addgene, Cambridge, MA, USA). Cells were transfected with either TOPFlash or FOPFlash (100 ng) and the internal control plasmid pRL–TK (5 ng; Promega), using ViaFect™ Transfection Reagent (Promega) in 96-well plates at 2 × 10^4^ cells per well. The cells were incubated for 24 h, and lysed to measure luciferase reporter gene expression by Luciferase Assay System (E1500, Promega). Firefly luciferase activity was normalized to Renilla luciferase activity. All results are expressed as a mean ± SEM of independent quintuplicate cultures. To test the secretion of WNTs from KIF3A shRNA cells, HEK293T cells were seed in 96-well plates at 2 × 10^4^ cells per well. Cells were transfected with either TOPFlash or FOPFlash (100 ng) and the internal control plasmid pRL-TK (5 ng; Promega) using ViaFect™ Transfection Reagent (Promega). The next day, the medium was changed to growth medium or conditioned medium from KIF3A knockdown cells treated without or with IWP-2. After 48 hr, WNT3A was treated. The next day, luciferase reporter gene expression was measured by Luciferase Assay System. All results are expressed as a mean ± SEM of independent quintuplicate cultures.

### Cell proliferation experiment

Cell proliferation was monitored every 24 h for 4 days using a Cell Counting Kit-8 (CCK-8) assay (Dojindo Laboratories, Kumamoto, Japan). Cells were plated in 96-well plates at 3 × 10^3^ cells per well. CCK-8 was added to each well, and cells were incubated at 37 °C for 2 h. Absorbance was measured at 450 nm.

### Human NSCLC xenograft tumour model

The entire experimental protocol was conducted in compliance with the institutional guidelines and approved by *Institutional Animal Care and Use Committee* (*IACUC*) *of the Asan Institute for Life Sciences, Asan Medical Center, Korea*. To generate xenograft tumour models, 100 μl of SW900 cells transduced with either NT-sh or KIF3A sh2 (6 × 10^6^ cells/100 μl) mixed in Matrigel (Corning, NY) were subcutaneously injected into BALB/cA-nu nude mice (6 weeks old). After 12 days, tumour growth was measured using calipers. The estimated volume was calculated according to the formula: Tumour volume (*mm*^3^) = 0.5 × *length* × *width*^2^.

### Sphere formation assay

To obtain tumour spheres in culture, cells were plated in ultra-low attachment dishes (Corning) at a density of 1 × 10^4 ^cells/ml in serum-free medium (DMEM/F12; Lonza, Basel, Switzerland) supplemented with 20 ng/ml of basic fibroblast growth factor (bFGF, Invitrogen), 50 ng/ml human epidermal growth factor (hEGF, Invitrogen), N2 (Invitrogen), B27 (Invitrogen), 10 μM Rock inhibitor (Enzo life science) and 1% penicillin/streptomycin (Gibco). Culture medium was replaced twice a week.

### Transwell migration assay

Transwell chambers (Corning) were used according to the manufacturer’s instructions. Briefly, RPMI 1640 containing 10% FBS was added to each bottom chamber. Then, the top chambers, which have uncoated transwell membrane filter inserts (6.4 mm in diameter, 8 um pore size), were placed in each well of a 24 well culture plate (Thermo Fisher Scientific). Cells (A549: 5 × 10^4 ^cells/200 μl, SW900: 1 × 10^5^ cells/200 μl) were pipetted into the top chambers containing RPMI 1640 without FBS. After 24 h incubation, non-migrating cells were removed from the upper face of the filter using cotton swabs and cells on the lower filter surface were fixed in 4% PFA for 15 min. After washed using PBS, the cells were stained with haematoxylin (Sigma). The number of cells per microscopic field was counted light microscopically. Migration capacity was measured by OD value and the average OD value of migrating cells within five random fields was calculated.

### Immunoprecipitation

For immunoprecipitation reactions, cells were lysed in lysis buffer (Cell Signaling Technology) containing phosphatase inhibitor cocktail C (Santa Cruz). The concentration of proteins in cell lysates was quantified by the Enhanced BCA Protein Assay Kit (Pierce Biotechnology, Inc.). Each 5 ul antibody for β-arrestin (1:200, BioLegend Inc.) or axin (1:200, Cell Signaling Technology) per 1 mg lysate was incubated overnight at 4 °C with constant rotation. The next day, a protein G sepharose (GE Healthcare Bio-Sciences Corp.) bead was added to each lysate for 4 h at 4 °C with constant rotation. Unbound proteins were removed by four washes using lysis buffer and proteins were eluted from the beads by the addition of 40 μl sample buffer. SDS-PAGE was used to analyze 20 μl samples.

### Immunohistochemistry

Samples were formalin-fixed, paraffin-embedded, sectioned, and stained with following primary antibodies as previously described^53^. The samples were incubated with primary antibodies including anti-KIF3A (1:750; Abcam), anti-β-catenin (1:1000; Cell Signalling Technology), and anti-cyclin D1 (1:1000; Cell Signalling Technology). The sections were subsequently incubated with secondary antibodies and then visualized using an ultraView Universal DAB Detection kit (Ventana Medical Systems). Nuclei were counterstained with Harris hematoxylin.

### Primary tumour cell preparation

The entire experimental protocol was conducted in compliance with the institutional guidelines. The study protocol was approved by *the Ethics Committee of Asan Medical Center, Korea.* Surgical samples were collected from consenting patients at the Asan Medical Center. All subjects approved the “informed consent” to the study. All patients were first diagnosed with primary NSCLC and showed metastasis. Samples were shipped to the laboratory in cold Hank’s Balanced Salt Solution (HBSS) with antibiotics (Lonza) within 1 h of removal from patients. Samples were washed with cold HBSS with antibiotics three times, chopped with a sterile blade, and incubated in 0.001% DNase (Sigma-Aldrich), 1 mg/ml collagenase/dispase (Roche, Indianapolis, IN), 200 U/ml penicillin, 200 mg/ml streptomycin, and 0.5 mg/ml amphotericin B (2% antibiotics, Sigma) in DMEM/F12 medium (Lonza) at 37 °C for 2 h with intermittent shaking. After incubation, the suspensions were repeatedly triturated and passed through 70-μm cell strainers (BD Falcon, San Jose, CA). The strained cells were centrifuged at 1000 rpm for 3 min. Cells were seeded in Collagen-I coated dishes (Corning) and cultured in serum-free medium (DMEM/F12; Lonza) supplemented with 20 ng/ml of basic fibroblast growth factor (Invitrogen), 50 ng/ml human epidermal growth factor (Invitrogen), N2 (Invitrogen), B27 (Invitrogen), 10 μM ROCK inhibitor (Enzo Life Sciences), and 1% penicillin/streptomycin (Gibco).

### Statistical analysis

Data are mainly expressed as a mean ± SEM from at least three experiments. Statistical analysis was performed with SPSS and graphs were designed using Graph Pad Prism 5. *P* < 0.05 was considered to indicate a statistically significant difference.

## Additional Information

**How to cite this article**: Kim, M. *et al*. KIF3A binds to β-arrestin for suppressing Wnt/β-catenin signalling independently of primary cilia in lung cancer. *Sci. Rep.* **6**, 32770; doi: 10.1038/srep32770 (2016).

## Supplementary Material

Supplementary Information
