# Corrigendum: KIF3A binds to β-arrestin for suppressing Wnt/β-catenin signalling independently of primary cilia in lung cancer

**DOI:** 10.1038/srep46773

**Published:** 2017-04-25

**Authors:** Minsuh Kim, Young-Ah Suh, Ju-Hee Oh, Bo Ra Lee, Joon Kim, Se Jin Jang

Scientific Reports 6: Article number: 3277010.1038/srep32770; published online: 09 06 2016; updated: 10 20 2015

The Acknowledgments section of this Article contains a typographical error, where:

“Ministry of Science, ICT & Future Planning (MSIP) (#NRF-2011-0013927)”

should read

“Ministry of Science, ICT & Future Planning (MSIP) (#NRF-2011-0030105)”.

