# Supplementary material for: KIF3A binds to β-arrestin for suppressing Wnt/β-catenin signalling independently of primary cilia in lung cancer
Source: Sci Rep. 2016 Sep 6;6:32770. doi: 10.1038/srep32770 (PMC5011747; doi:10.1038/srep32770)
Supplement: Supplementary Information [file srep32770-s1.pdf]

Supplementary information for

KIF3A binds to β-arrestin for suppressing Wnt/β-catenin signalling independently of primary cilia in lung cancer

Minsuh Kim[1,3], Young-Ah Suh[3], Ju-Hee Oh[3], Bo Ra Lee[3], Joon Kim[1,2*] and Se Jin Jang[3,4*]

[1] Biomedical science and engineering interdisciplinary program, KAIST, Daejeon, South Korea

[2] Graduate School of Medical Science and Engineering, KAIST, Daejeon, South Korea

[3] Asan Institute for Life Sciences, University of Ulsan College of Medicine, Asan Medical Center, Seoul, South Korea

[4] Asan Center for Cancer Genome Discovery, Department of Pathology, University of Ulsan College of Medicine, Asan Medical Center, Seoul, South Korea

[*] Corresponding Authors:
Se Jin Jang, MD, PhD & Joon Kim, PhD.

Supplementary materials and methods

Annexin-V assay

For apoptosis analyses, we used an annexin V assay (Invitrogen). Cells treated with HPI4 were washed in ice-cold PBS without Ca^{2+} or Mg^{2+}. We then resuspended 1×10^6 cells in 1 ml of binding buffer and 200 µl of this cell suspension was incubated with 2 µl of 100 µg/ml propidium iodide and 10 µl of Alexa Fluor® 488 annexin V for 15 min in the dark at room temperature. Flow cytometric analysis was immediately performed using a FACS Calibur Instrument (Becton Dickinson).

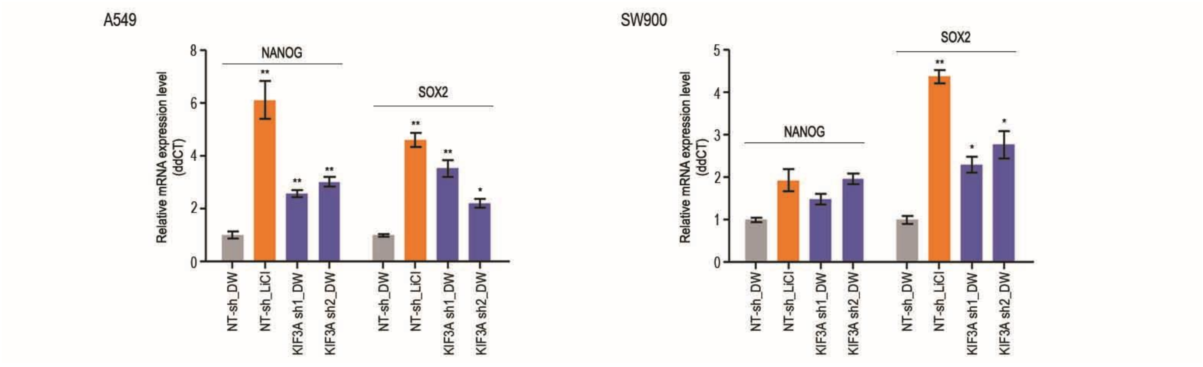

Supplementary Figure 1. LiCl increases stemness marker expression in NT-sh cells. Quantitative RT-PCR analysis confirming stemness marker expression in NT-sh cells treated to LiCl (20 mM) for 24 h. β-actin was used as an internal control for quantitative RT-PCR (n = 3; error bars indicate SEM; p-value was determined by one-way ANOVA). *, $P < 0.05$; **, $P < 0.01$.

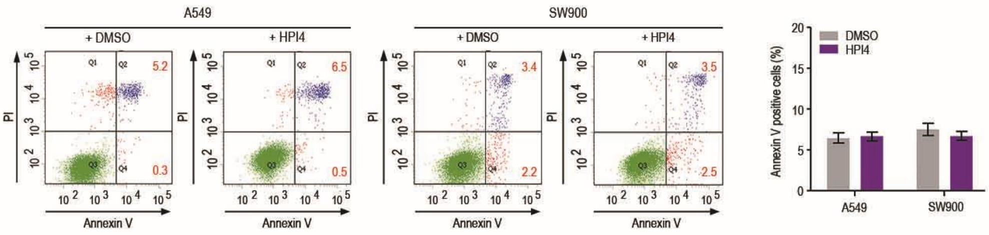

Supplementary Figure 2. HPI4 does not induce apoptosis. Annexin-V assay to examine apoptosis in cells treated with HPI4 (20 μM). *, $P < 0.05$; **, $P < 0.01$.

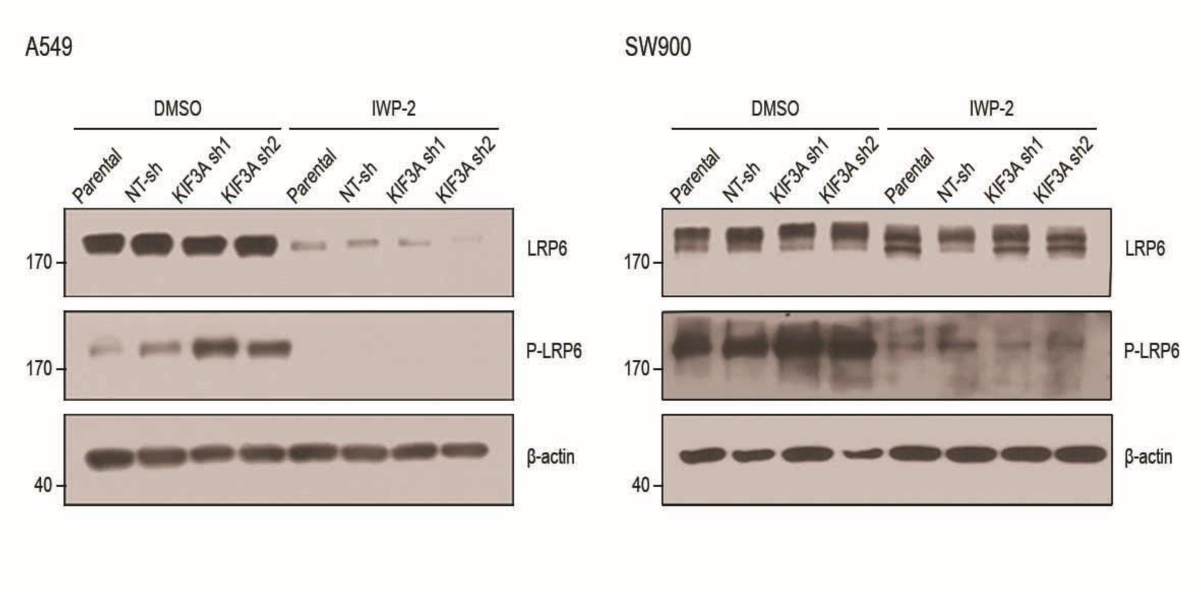

Supplementary Figure 3. IWP-2 suppresses LRP6 and P-LRP6 expression in KIF3A knock down cells. Immunoblot analysis showing protein levels of LRP6 and P-LRP6 after treating IWP-2 (30 μM) for 72 h. β-actin was used as a loading control. Full-length blot is shown in the supplementary Figure 5, and the gels have been run under the same experimental conditions.

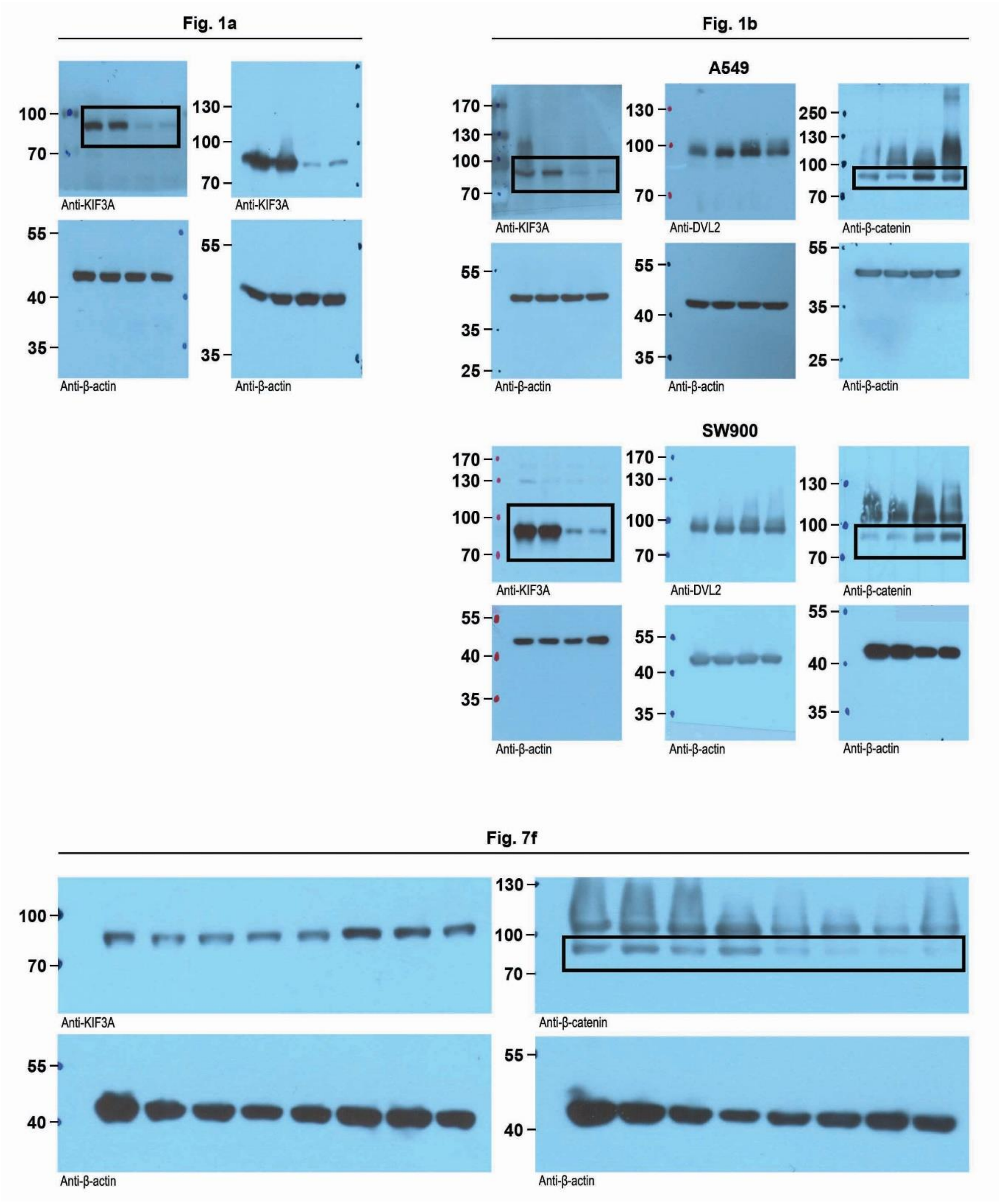

Supplementary Figure 4. Full-length blots in this study.

Fig. 6a

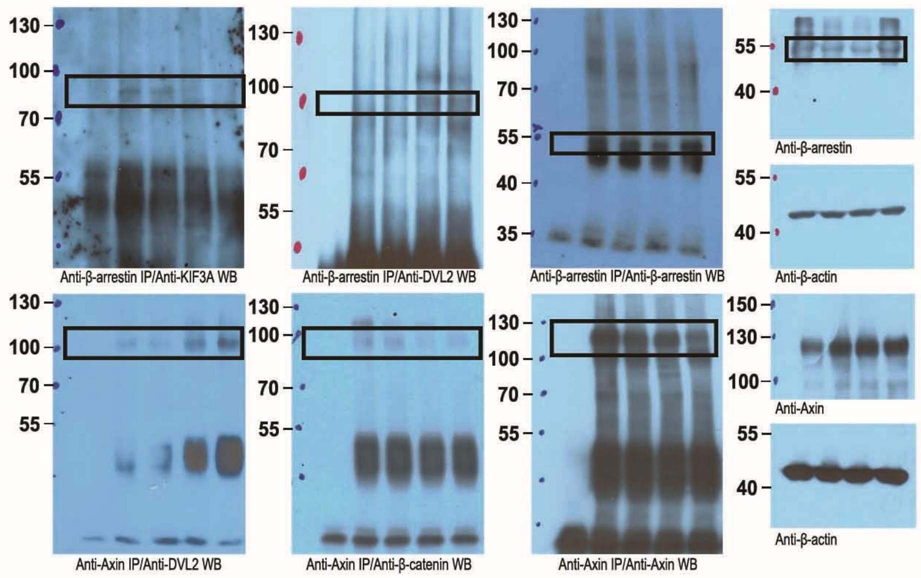

Fig. 6b

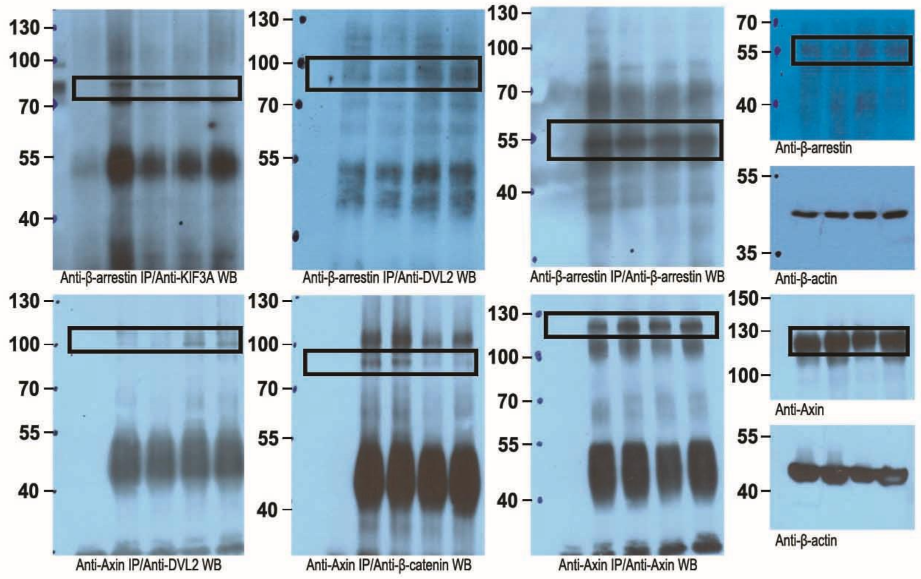

Supplementary Figure 3

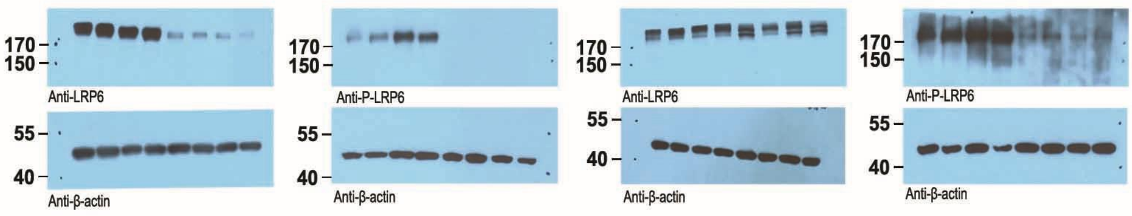

Supplementary Figure 5. Full-length blots in this study.

Supplementary Table 1. KIF3A, β-catenin and Cyclin D1 expression scores according to IHC analysis.

Case #	Sex	Age	S.M.K.	KIF3A			β-catenin			CycD1			1: Adenocarcinoma, 2: Squamous cell carcinoma, 3: Adenosquamous cell carcinoma, 4: Sarcomatoid carcinoma, 5: Large cell carcinoma
				P	R1	R2	P	R1	R2	P	R1	R2	
1	F	48		1	1		4	4		45	45		1
2	M	32		0	0	1	2	3	1	30	25	10	2
3	M	79		0	1		2	2		30	1		1
4	F	54		0	2		3	3		25	65		1
5	M	70		1	2		1	1		70	55		1
6	M	61		1	2		2	2		1	1		1
7	M	54		1	1		1	1		5	5		1
8	M	52		1	2		2	1		5	10		2
9	M	56		0	0		2	2		85	75		1
10	M	40		1	1	2	1	1	4	70	5	25	1
11	F	55		2	1		3	2		10	40		1
12	M	55		1	0		1	4		80	5		5
13	F	53		2	0		1	3		5	55		1
14	M	36		0	2		3	1		35	1		2
15	M	68		0	1		4	3		1	1		5*
16	F	45		1	1		4	3		60	1		1
17	M	57		0	0		3	3		35	95		2
18	M	68		1	0		1	3		10	65		1
19	F	42		0	2	0	1	4	4	15	40	20	1
20	M	53		1	1		2	2		80	75		1

**21**	**M**	**71**		0	1		3	2		40	5		**2**
**22**	**F**	**68**		1	0		3	3		25	10		**1***
**23**	**M**	**37**		1	0		2	2		85	65		**1**
**24**	**M**	**46**		2	1	0	2	3	3	1	1	1	**2**
**25**	**F**	**58**		2	n.d.		4	n.d.		75	n.d.		**3**
**26**	**M**	**65**		0	2		2	1		20	30		**2**
**27**	**M**	**56**		1	1		2	1		10	5		**1**
**28**	**M**	**55**		0	1		3	3		15	15		**2**
**29**	**M**	**64**		0	1		3	3		25	15		**2**
**30**	**M**	**51**		1	0		2	4		60	40		**1**
**31**	**M**	**43**		0	1	1	3	2	3	35	50	35	**2**
**32**	**M**	**61**		0	0	0	3	4	3	45	40	25	**2**
**33**	**M**	**59**		2	1		4	4		35	65		**2**
**34**	**M**	**62**		2	n.d.		0	n.d.		1	n.d.		**1**
**35**	**F**	**38**		0	1		3	2		25	30		**1**
**36**	**M**	**52**		1	1		1	1		40	40		**2**
**37**	**M**	**68**		1	0		1	2		80	15		**1**
**38**	**M**	**60**		0	1		4	1		70	30		**2**
**39**	**M**	**58**		2	1	2	2	2	2	15	15	25	**1**
**40**	**M**	**61**		0	0		4	3		85	75		**2**
**41**	**M**	**67**		2	2	2	2	3	3	20	60	55	**1**
**42**	**M**	**53**		0	1		2	2		40	65		**2**
**43**	**M**	**65**		0	0		2	2		35	25		**4**
**44**	**F**	**53**		1	2		4	2		80	50		**1**

**45**	**M**	**51**		1	n.d.		3	n.d.		1	n.d.		**1**
**46**	**M**	**69**		1	1		2	3		40	25		**2**
**47**	**F**	**67**		2	2		3	4		50	65		**1**
**48**	**M**	**56**		0	n.d.		4	n.d.		55	n.d.		**1**
**49**	**M**	**61**		1	2		1	2		5	40		**2**
**50**	**M**	**65**		2	2		3	3		15	25		**1**
**51**	**M**	**60**		0	1		1	1		20	5		**2**
**52**	**M**	**71**		1	0		4	4		45	75		**2**
**53**	**M**	**71**		0	n.d.		3	n.d.		55	n.d.		**2**
**54**	**M**	**73**		2	2		2	1		15	0		**2**
**55**	**M**	**60**		2	2		2	4		35	25		**1**
**56**	**M**	**72**		1	1		2	2		35	5		**1**
**57**	**M**	**70**		2	2		1	1		5	5		**1**
**58**	**F**	**38**		2	1		1	1		1	30		**1**
**59**	**M**	**45**		1	2		4	3		45	50		**1**
**60**	**M**	**68**		2	1	1	1	2	2	45	5	50	**1**
**61**	**F**	**46**		0	2		3	2		55	40		**1**
**62**	**M**	**53**		0	2		2	2		1	1		**2**
**63**	**F**	**70**		1	1		1	1		10	10		**1**
**64**	**M**	**54**		1	1		4	4		60	85		**1**
**65**	**M**	**70**		1	0		2	3		45	5		**2**
**66**	**M**	**50**		0	0		2	4		60	70		**2**
**67**	**M**	**71**		0	2		3	1		15	70		**2**
**68**	**M**	**64**		2	1		2	2		15	10		**1**

**69**	**M**	**54**		2	2		2	2		25	30		**1**
**70**	**M**	**51**		2	2	0	2	2	3	5	5	1	**1**
**71**	**F**	**60**		2	2		2	3		30	10		**1**
**72**	**F**	**62**		0	1		3	2		25	45		**1**
**73**	**F**	**45**		0	2		3	3		40	35		**1**
**74**	**F**	**61**		0	1		3	3		50	65		**1**
**75**	**M**	**51**		2	2	2	3	2	2	55	95	75	**1**
**76**	**M**	**50**		2	2		2	3		15	60		**1**
**77**	**M**	**60**		2	1		4	4		55	50		**2***
**78**	**M**	**57**		2	1		2	3		45	85		**1**
**79**	**M**	**60**		2	1		1	2		15	50		**1**
**80**	**M**	**56**		0	2		3	3		30	35		**1**
**81**	**M**	**59**		2	0		1	1		15	5		**1**
**82**	**F**	**37**		0	1	1	4	3	2	85	25	60	**1**
**83**	**F**	**61**		1	1	1	2	2	2	1	5	15	**1**
**84**	**M**	**75**		2	2		2	4		1	25		**2**
**85**	**M**	**66**		2	2		1	1		15	30		**1**
**86**	**F**	**63**		1	1		1	1		25	50		**1**
**87**	**M**	**68**		2	2		1	2		5	35		**1**
**88**	**F**	**58**		0	0		2	3		5	1		**1**
**89**	**M**	**74**		1	2	2	2	2	3	45	55	15	**2**
**90**	**M**	**57**		1	1		2	2		70	60		**1**
**91**	**M**	**56**		1	2		3	2		80	100		**2**
**92**	**M**	**57**		2	2		1	2		25	10		**1**

**93**	**M**	**68**		0	1		1	2		10	15		**2**
**94**	**M**	**60**		1	n.d.		2	n.d.		60	n.d.		**2**
**95**	**M**	**66**		2	0		2	0		15	5		**2**
**96**	**F**	**67**		1	0		2	2		30	15		**1**
**97**	**M**	**63**		2	2		2	3		10	20		**2**
**98**	**F**	**64**		2	2		2	1		5	25		**1**
**99**	**F**	**56**		2	2		3	2		5	25		**1**
**100**	**M**	**54**		2	2		2	3		10	10		**1**
**101**	**M**	**61**		0	2		3	2		75	5		**2**
**102**	**M**	**65**		0	1		3	2		70	10		**2**
**103**	**M**	**58**		2	2	0	1	1	2	1	1	20	**1**
**104**	**F**	**44**		2	2		1	3		5	10		**1**
**105**	**M**	**75**		1	1	0	1	3	2	10	50	25	**2**
**106**	**F**	**50**		1	1		1	3		20	35		**1**
**107**	**M**	**71**		2	2		4	4		5	5		**2**
**108**	**M**	**55**		2	0	2	1	2	2	5	25	25	**1**
**109**	**F**	**76**		1	2		4	4		85	20		**1**
**110**	**M**	**66**		1	1		2	1		20	65		**2**

n.d; IHC data not done, M; male, F; female, S.M.K. (Smoking status): dark gray; ever smoker, empty; never smoker. P; primary, R1; 1st recurred tumors, R2; 2nd recurred tumors, score 4 in β-catenin score; nuclear expression, n*; recurred tumor showed different histologic feature with primary tumor.

Supplementary Table 2. Clinic characteristics of patient-derived primary NSCLC cells.

PCC #	Sex	Age	Histology
PCC1	58	F	Adenocarcinoma
PCC2	60	F	Adenocarcinoma
PCC3	60	F	Sarcomatoid carcinoma
PCC4	72	M	Squamous cell carcinoma
PCC5	66	M	Squamous cell carcinoma
PCC6	54	M	Squamous cell carcinoma
PCC7	61	M	Adenocarcinoma
PCC8	72	F	Adenocarcinoma

Supplementary Table 3. The list of primer sequences used for quantitative RT-PCR and RT-PCR.

Gene	Primer name	Primer sequence
NANOG	NANOG_f	5'-CTAAGAGGTGGCAGAAAAACA-3′
	NANOG_R	5'-CTGGTGGTAGGAAGAGTAAAGG-3′
OCT4	OCT4_f	5'-GACAACAATGAAAATCTTCAGGAGA-3′
	OCT4_R	5'-CTGGCGCCGGTTACAGAACCA-3
SOX2	SOX2_f	5'-ACGACGTGAGCGCCCTGCAGTACAA-3'
	SOX2_R	5'-GCTGGAGCTGGCCTCGGACTTGACC-3'
β-actin	β-actin_f	5'-AGAGCTACGAGCTGCCTGAC-3'
	β-actin_R	5'-AGCACTGTGTTGGCGTACAG-3'
GAPDH	GAPDH_f	5'-AGCCACATCGCTCAGACAC-3'
	GAPDH_R	5'-GCCCAATACGACCAAATCC-3'
